# Pyroptosis-immune cell cross talk in asthma: From molecular mechanisms to precision therapeutics

**DOI:** 10.3389/fimmu.2025.1727122

**Published:** 2025-11-28

**Authors:** Feng-Xian Ni, Hui-Xian Wang, Pei-Sheng Chen, Hua-Jing Huang, Hui-Hui Chen, Dong-Hui Huang, Ze-Bo Jiang

**Affiliations:** 1 Zhuhai Hospital of Integrated Traditional Chinese and Western Medicine, Zhuhai, Guangdong, China; 2 Department of Respiratory Medicine, Guangdong Provincial Hospital of Traditional Chinese Medicine Zhuhai Branch, Zhuhai, Guangdong, China; 3 Northeastern University, Boston, MA, United States

**Keywords:** pyroptosis, asthma, inflammasome, gasdermin D, eosinophils, IL-33, airway remodeling, NLRP3

## Abstract

Asthma is a heterogeneous chronic airway disease characterized by complex inflammation. Pyroptosis, a pro-inflammatory form of programmed cell death mediated by gasdermin (GSDM) family proteins, has recently emerged as a critical amplifier of airway inflammation and tissue remodeling in asthma. This review delineates the molecular underpinnings of pyroptosis, focusing on the roles of canonical (e.g., NLRP3-caspase-1) and non-canonical (e.g., caspase-4/5/11) inflammasome pathways, as well as the broader concept of PANoptosis. We elaborate on how the pore-forming activity of GSDMD and other GSDMs facilitates the release of potent pro-inflammatory cytokines (IL-1β, IL-18), driving pathogenic cross talk among structural cells (epithelium), innate immune cells (macrophages, eosinophils, ILC2s), and adaptive immunity. Crucially, we contextualize pyroptosis within distinct asthma endotypes, proposing that allergen-driven, NLRP3-dominated pathways may underpin Th2-high/eosinophilic inflammation, while pollutant/viral-triggered, non-canonical/AIM2 pathways may favor Th2-low/neutrophilic phenotypes. The translational potential of targeting pyroptosis is underscored through a discussion of biomarkers (e.g., GSDMD-N, IL-18) and a comprehensive summary of preclinical and early clinical inhibitors targeting NLRP3, GSDMD, and key cytokines. By synthesizing these multifaceted roles, this review posits that a nuanced understanding of pyroptosis networks holds significant promise for pioneering endotype-specific therapeutic strategies in asthma management.

## 1 Introduction

Asthma represents a pervasive global health challenge, afflicting millions with its hallmark symptoms of wheezing, breathlessness, and variable airflow obstruction. The traditional view of asthma as a single disease entity has been superseded by the paradigm of heterogeneity, encompassing diverse clinical, physiological, and inflammatory phenotypes (1). Central to this complexity are distinct molecular endotypes, broadly classified as T-helper 2 (Th2)-high and Th2-low, which govern the underlying immune pathology and therapeutic responsiveness (2, 3). Th2-high asthma, characterized by elevated levels of IL-4, IL-5, and IL-13 alongside eosinophilic infiltration, typically demonstrates favorable responses to corticosteroids and specific biologics (4). In contrast, Th2-low asthma, often featuring neutrophilic inflammation, is frequently associated with severe disease and diminished sensitivity to conventional therapies (5), underscoring the pressing need for novel therapeutic avenues.

Recent investigations have spotlighted the crucial role of airway epithelial injury in asthma pathogenesis. The epithelium acts not only as a physical barrier but also as an active immunological sentinel. Upon exposure to environmental insults such as allergens, pollutants, and viruses, compromised epithelial cells release a cascade of pro-inflammatory mediators and damage-associated molecular patterns (DAMPs), thereby perpetuating chronic inflammation and instigating structural airway remodeling (6).

Pyroptosis, a lytic form of programmed cell death executed by gasdermin (GSDM) family proteins, has emerged as a pivotal mechanism linking environmental triggers to dysregulated immune responses in asthma (7). Activation of pyroptotic pathways in both structural and immune cells by allergens, viruses, or pollutants culminates in GSDMD-mediated pore formation, cellular lysis, and the release of mature IL-1β and IL-18 (8). These events recruit and activate additional immune cells, thereby amplifying the inflammatory cascade. The breach of the epithelial barrier through lytic cell death further exacerbates disease pathology. Supporting this, elevated markers of pyroptosis are detected in asthma patients, and GSDMD activation correlates with increased airway hyperresponsiveness (AHR) in experimental models (9), suggesting its contribution to disease severity. While Th2-high asthma is driven by cytokines like IL-4, IL-5, and IL-13 and often responds well to corticosteroids and biologics, the pathogenesis and treatment of Th2-low, neutrophilic, or pauci-granulocytic asthma remain less defined and often exhibit steroid resistance (10).

Nevertheless, the precise involvement of pyroptosis in asthma pathophysiology and its interplay with other cell death modalities remain incompletely elucidated. This review aims to address these gaps by critically examining the contribution of pyroptosis to asthma development and progression. We will dissect the molecular mechanisms, explore its impact on inflammation and barrier function, and investigate the therapeutic potential of targeting pyroptosis to improve clinical outcomes, particularly in severe or treatment-refractory asthma. Advancing our comprehension of pyroptosis within the intricate network of asthma pathogenesis may pave the way for innovative and more effective treatment modalities.

## 2 Molecular mechanisms of pyroptosis in asthma

**Figure 1:**
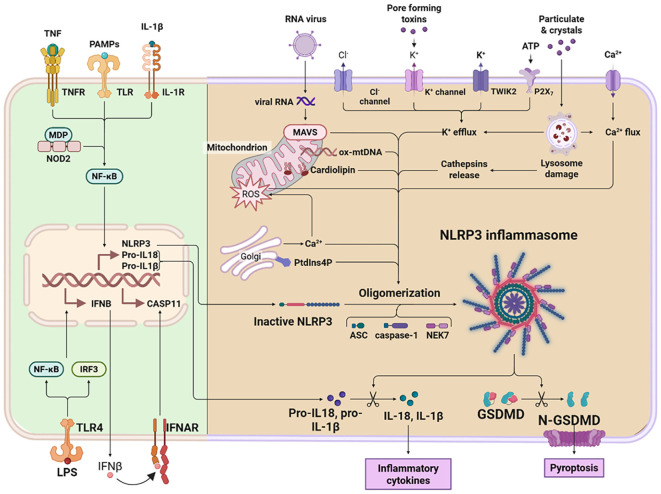
An overview of the molecular mechanisms of pyroptosis. Pyroptosis is a form of programmed cell death mediated primarily by inflammatory caspases. The canonical pathway is driven by caspase-1 activation. Upon recognition of cytosolic pathogen-associated molecular patterns (PAMPs) or damage-associated molecular patterns (DAMPs), inflammasome complexes assemble, leading to the proteolytic activation of caspase-1. Active caspase-1 then cleaves pro-IL-1β and pro-IL-18 into their biologically active forms and also cleaves gasdermin D (GSDMD). The non-canonical pathway, in contrast, is predominantly mediated by caspase-11 in mice and its homologs caspase-4 and caspase-5 in humans. Activation of these caspases generally occurs independently of pattern recognition receptor (PRR)–mediated inflammasome signaling. Instead, caspase-11 directly binds intracellular lipopolysaccharide (LPS), prompting its oligomerization and activation. This in turn proteolytically cleaves and activates GSDMD, ultimately disrupting membrane integrity. GSDMD contains two conserved domains: an N-terminal pore-forming effector domain and a C-terminal autoinhibitory domain. The N-terminal domain is responsible for executing pyroptosis, whereas the C-terminal domain maintains autoinhibition. Under resting conditions, these domains are connected via a flexible linker region. Upon stimulation, caspase-1/4/5/11 cleaves GSDMD, releasing its N-terminal fragment (GSDMD-NT). This fragment binds to membrane phospholipids, oligomerizes, and forms transmembrane pores. These pores compromise plasma membrane integrity, resulting in osmotic lysis and pyroptotic cell death. Concurrently, they facilitate the release of mature inflammatory cytokines such as IL-1β and IL-18, thereby amplifying the inflammatory cascade.

Pyroptosis is a lytic, highly inflammatory form of programmed cell death distinct from apoptosis due to its dependence on GSDM family proteins and the robust release of pro-inflammatory cytokines, including IL-1β and IL-18 (11). In asthma, this pathway is not merely a passive consequence but an active driver of pathology, amplifying airway inflammation, compromising epithelial barrier integrity, and promoting aberrant tissue remodeling (12). The core machinery involves pathogen- and danger-sensing inflammasomes, inflammatory caspases, and the pore-forming GSDM proteins (13). A comprehensive understanding of these molecular pathways is essential for identifying novel therapeutic targets to disrupt the self-perpetuating inflammatory cycles characteristic of severe and refractory asthma. An overview of the molecular mechanisms of pyroptosis is shown in **Figure 1**.

### 2.1 Triggers initiating pyroptosis in asthmatic airways

**Figure 2:**
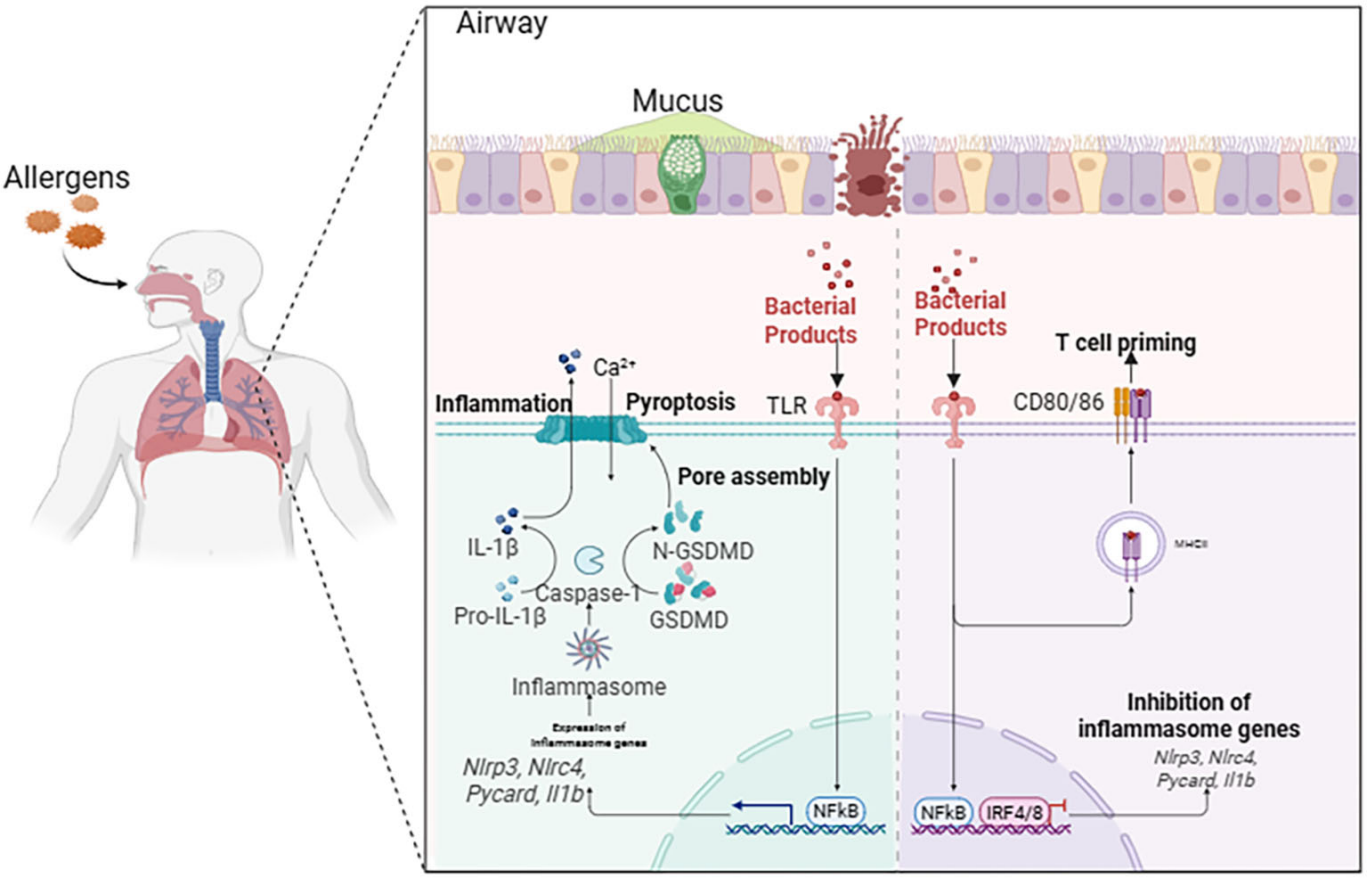
Triggers initiating pyroptosis in asthmatic airways. In the airways of asthma, environmental allergens, respiratory viral infections, and inhaled air pollutants can cause pyroptosis of cells, simultaneously activating pyroptosis-related pathways. These signaling pathways converge on the activation of inflammasomes within airway epithelial cells, macrophages, and other resident immune cells, thereby exacerbating the severity and deterioration of asthma.

The asthmatic airway presents a unique microenvironment where diverse exogenous and endogenous stimuli converge to activate pyroptotic cell death (**Figure 2**) (11). These triggers engage specific molecular sensors that nucleate the assembly of inflammasomes, initiating a proteolytic cascade culminating in GSDM cleavage. The formation of GSDMD pores in the plasma membrane leads to cell swelling, lytic death, and the extracellular release of pro-inflammatory cytokines and damage-associated molecular patterns (DAMPs), thereby profoundly amplifying local and systemic immune responses (14). The major triggers—environmental allergens, respiratory viruses, and airborne pollutants—engage distinct yet occasionally overlapping signaling pathways that converge on inflammasome activation within airway epithelial cells, macrophages, and other immune effector cells, significantly aggravating disease severity and exacerbation frequency (**Table 1**) (15).

**Table 1:** Molecular pathways linking environmental triggers to pyroptosis in asthma.

Trigger	Molecular sensor	Key signal	Downstream effectors	Pathogenic outcome	References
HDM (Der p 1)	TLR4 → NLRP3	K^+^ efflux	Caspase-1, GSDMD	Th2 inflammation, IL-33 release	(17)
Rhinovirus/RSV	RIG-I → NLRP3	mtROS	Caspase-1, GSDMD	Viral exacerbations, IL-1β storm	(30)
Viral RNA	Caspase-4/5	Cytosolic viral RNA	GSDMD	Epithelial barrier disruption	(31)
PM2.5	AIM2	Oxidative stress/dsDNA	Caspase-1, GSDMD	Neutrophilic inflammation	(32)

#### 2.1.1 Allergen proteases as initiators of inflammasome activation

Protease allergens from house dust mites (HDM), such as the cysteine protease Der p 1, are pivotal in initiating and sustaining airway inflammation and remodeling. Der p 1 directly interacts with airway epithelial cells and macrophages, enzymatically cleaving epithelial tight junctions and compromising barrier integrity (16). This disruption facilitates potassium ion (K^+^) efflux, a key cellular event for NLRP3 inflammasome priming and activation (17). The efflux promotes NLRP3 inflammasome assembly and caspase-1 activation. Active caspase-1 then cleaves pro-IL-1β and pro-IL-33 into their mature, bioactive forms and processes GSDMD to initiate pyroptotic pore formation (17). The release of IL-1β and IL-33 creates a pro-inflammatory niche that potently enhances type 2 immune responses via recruitment and activation of Th2 cells and eosinophils, leading to airway hyperreactivity (AHR) and chronic inflammation. Preclinical *in-vivo* studies demonstrate that NLRP3 inhibition or genetic ablation significantly attenuates IL-1β secretion and AHR in HDM-challenged murine models, reducing airway inflammation and hyperreactivity by over 60% (18). Furthermore, Der p 1–mediated damage induces oxidative stress and modulates alarmins, establishing a feed-forward inflammatory amplification loop.

#### 2.1.2 Viral infections as potent amplifiers of pyroptosis

Respiratory viral infections, particularly by rhinovirus (RS) and respiratory syncytial virus (RSV), are major triggers of pyroptosis and frequent causes of acute asthma exacerbations (19). Viral RNA is detected by cytosolic sensors like RIG-I and MDA5, initiating NF-κB–driven transcriptional priming for pro-IL-1β and NLRP3 components (20). These viruses potently induce mitochondrial reactive oxygen species (mtROS) in infected airway epithelial cells, which facilitate NLRP3 inflammasome oligomerization and activation (21). This leads to caspase-1 activation, pyroptosis, and release of IL-1β and IL-18, amplifying inflammation and recruiting immune effector cells (39). Notably, cytoplasmic viral components can also activate the non-canonical inflammasome pathway, engaging caspase-4/5 in humans (caspase-11 in mice), which cleaves GSDMD independently of NLRP3, triggering further epithelial barrier disruption and exacerbating viral-induced asthma flare-ups (22). The clinical relevance of this pathway is underscored by findings of upregulated caspase-4 and GSDMD in bronchial biopsies from severe asthma patients with recurrent viral exacerbations (23).

#### 2.1.3 Airborne pollutants and oxidative stress-mediated inflammasome activation

Exposure to fine particulate matter (PM2.5) is a significant environmental risk factor. These pollutants induce oxidative stress and genomic damage in airway cells, generating cytoplasmic double-stranded DNA (dsDNA) fragments that serve as endogenous DAMPs (24). This dsDNA can activate the AIM2 inflammasome, which recruits the adaptor protein ASC and pro-caspase-1 to form an active complex. AIM2 activation culminates in caspase-1-mediated GSDMD cleavage, pyroptosis, and IL-18 release. IL-18 acts as a potent inducer of IFN-γ production by NK and T cells, perpetuating chronic inflammation (25). *In-vitro* studies using human airway epithelial cells exposed to PM_2.5_ show upregulated AIM2 expression and increased pyroptosis markers, effects that can be mitigated by antioxidants like N-acetylcysteine, confirming oxidative stress as a fundamental upstream regulator (26). Epidemiological studies consistently correlate high PM_2.5_ levels with increased asthma incidence and exacerbation rates, positioning the AIM2 inflammasome as a potential therapeutic target for pollutant-associated airway injury (27).

#### 2.1.4 Novel metabolic and post-translational regulators

Emerging research highlights the role of metabolic reprogramming and post-translational modifications in regulating pyroptosis. For instance, the metabolic enzyme Pyruvate Kinase M2 (PKM2) can translocate to the nucleus in its dimeric form upon stimuli such as poly-L-arginine (a mimic of eosinophil major basic protein), where it promotes NLRP3 transcription and subsequent inflammasome activation (28). This process is regulated by the mTORC1 signaling pathway. The PKM2-specific activator TEPP-46, which stabilizes PKM2 in its tetrameric form, can inhibit this nuclear translocation and subsequent pyroptosis, revealing a novel metabolic-inflammatory axis in asthma.

Furthermore, the ubiquitin-specific protease USP8 has been identified as a key negative regulator of pyroptosis. Its expression is downregulated following lipopolysaccharide (LPS) challenge in human bronchial epithelial cells (BEAS-2B). USP8 overexpression inhibits LPS-triggered inflammation and pyroptosis by activating the PI3K/AKT signaling pathway while concurrently inhibiting the NF-κB pathway, thereby attenuating the expression of NLRP3, GSDMD-N, caspase-1, IL-1β, and IL-18 (29).

#### 2.1.5 Integrative perspectives and future directions

Diverse triggers—allergens, viruses, pollutants—employ distinct molecular pathways (NLRP3, AIM2, non-canonical caspases) but converge on the common endpoint of GSDM-mediated pyroptosis and pro-inflammatory cytokine release. This convergence underscores inflammasomes as critical nodal points for therapeutic intervention (29). Inhibitors targeting specific inflammasome components, caspases, or the GSDM pores themselves hold promise for attenuating pyroptosis across multiple asthma phenotypes. Additionally, antioxidant therapies may broadly mitigate pollutant- and virus-driven inflammasome activation. Future research should leverage single-cell transcriptomics and proteomics to delineate cell type–specific inflammasome activation patterns and validate inflammasome-associated biomarkers in longitudinal clinical studies to inform personalized treatment approaches.

### 2.2 Principal pyroptotic pathways in asthma

Pyroptosis contributes to airway pathology through multiple, interconnected molecular cascades that modulate disease severity and help define distinct immune phenotypes. The central pyroptotic mechanisms include the canonical inflammasome pathway, the non-canonical caspase pathway, and the emerging, more integrated cell death program termed PANoptosis (33). The specific pathway activated is highly dependent on the nature of the environmental trigger and the responding cell type, ultimately driving different asthma endotypes.

#### 2.2.1 Canonical NLRP3 inflammasome pathway in Th2-high inflammation

The canonical NLRP3 inflammasome is the predominant driver of pyroptosis in allergen-induced, Th2-high asthma. This pathway requires a tightly regulated two-step activation process (34, 35). The priming signal (e.g., via TLR4) upregulates NLRP3 and pro-IL-1β via NF-κB (36). This signal (e.g., K^+^ efflux, mtROS) triggers NLRP3 oligomerization with ASC and procaspase-1 (37). Active caspase-1 cleaves GSDMD, liberating its pore-forming N-terminal domain (GSDMD-NT). This leads to pyroptotic cell lysis and the release of mature IL-1β and IL-18, which are critical for amplifying type 2 inflammation and AHR (31). Pharmacological blockade of NLRP3 using inhibitors such as MCC950 reduces airway inflammation in murine models of allergic asthma (38, 39), positioning it as a therapeutic target for Th2-high asthma.

#### 2.2.2 Non-canonical caspase-4/5–mediated pathway in Th2-low and viral exacerbations

The non-canonical pyroptotic pathway operates independently of inflammasome scaffolding and is implicated in Th2-low, neutrophilic asthma and viral exacerbations (40). This pathway is directly initiated when human caspase-4 and caspase-5 sense intracellular LPS or other microbial components (31). Upon binding its ligand, the caspase auto-activates and cleaves GSDMD, triggering pyroptosis. This pathway primarily disrupts the epithelial barrier and incites neutrophilic inflammation, contributing to the corticosteroid resistance often observed in severe neutrophilic asthma (41). Evidence from severe asthma patients reveals upregulated caspase-4 and GSDMD expression (40, 42, 43).

#### 2.2.3 Eosinophil granzyme A-driven pyroptosis

Severe eosinophilic asthma features an alternative, granzyme-mediated mechanism. Activated eosinophils release granzyme A (GZMA), which can enter airway epithelial cells. Inside the cell, GZMA activates caspase-3. Instead of leading to quiet apoptosis, activated caspase-3 cleaves gasdermin E (GSDME), shifting the mode of cell death to pyroptosis. The resulting GSDME-NT fragments form membrane pores, inducing lytic death and facilitating the release of the alarmin IL-33. This alarmin, in turn, recruits and activates more eosinophils and type 2 innate lymphoid cells (ILC2s), creating a feed-forward loop that exacerbates type 2 inflammation and remodeling. Genetic deletion of GSDME substantially reduces airway fibrosis and IL-33 secretion despite persistent eosinophilia, highlighting its pivotal role in driving pathology independent of eosinophil accumulation alone.

#### 2.2.4 PANoptosis: an integrated inflammatory cell death pathway

PANoptosis is a recently conceptualized, pro-inflammatory programmed cell death pathway that incorporates components and key features of pyroptosis, apoptosis, and necroptosis. It is regulated by multifaceted protein complexes called PANoptosomes (**Table 2**) (44). Triggers include viral RNAs, LPS, and DAMPs (e.g., ATP and mitochondrial DNA), which can activate sensors like Z-DNA binding protein 1 (ZBP1), AIM2, or RIPK1. Sensor engagement nucleates PANoptosome formation, recruiting molecular components from all three death pathways (e.g., NLRP3, caspase-1/8/3, RIPK3, MLKL), resulting in a potent, coordinated inflammatory cascade that is not easily defined by any single death pathway alone.

**Table 2:** Core components of PANoptosis pathways.

Pathway	Key effectors	Activation triggers	References
Pyroptosis	GSDMD, NLRP3, caspase-1	PAMPs (e.g., viral RNA)	(48)
Apoptosis	Caspase-3/7, BAX/BAK	DNA damage, oxidative stress	(49)
Necroptosis	RIPK3, p-MLKL	TNF receptor signaling	(50)
PANoptosis	ZBP1-PANoptosome complex	Pathogen sensors, DAMPs	(51)

In severe neutrophilic asthma, insults like viral infection can activate ZBP1, forming a ZBP1-based PANoptosome (45). Within this complex, caspase-8 can cleave GSDMD, inducing pore formation and IL-1β/IL-18 secretion, while also activating caspase-3 for GSDME cleavage. Concurrently, RIPK3 phosphorylates MLKL, inducing necroptotic membrane rupture and DAMP release (46, 47). This interplay creates a positive feedback loop, dramatically amplifying inflammation and tissue injury. Released DAMPs (e.g., ATP, HMGB1) activate macrophages and ILC2s, escalating the production of IL-5, IL-13, and IL-17, which orchestrates mixed granulocytic inflammation. Single-cell transcriptomics of severe asthma biopsies has revealed epithelial cells co-expressing GSDMD and MLKL, correlating with neutrophilia and impaired steroid signaling, indicating PANoptosis as a key contributor to refractory phenotypes.

### 2.3 Gasdermin family in asthma: beyond GSDMD

While GSDMD is the canonical executor, other gasdermin family members contribute unique roles in airway inflammation, remodeling, and genetic susceptibility (**Table 3**).

**Table 3:** Gasdermin proteins in asthma: expression, regulation, and clinical relevance.

Gasdermin	Primary site in lung	Activator	Key function in asthma	Therapeutic implication	References
GSDMD	Macrophages, Neutrophils	Caspase-1/4/5/11	IL-1β/IL-18 release, NETosis	Disulfiram blocks Cys191 palmitoylation	(52)
GSDME	Bronchial epithelium	Caspase-3, Granzyme	Apoptosis-to-pyroptosis shift, remodeling	Epithelial-specific inhibitors reduce fibrosis	(53)
GSDMB	Epithelium, T cells	Caspase-1 (partial)	Pro-inflammatory pore formation; sulfatide binding	Risk allele modulation (e.g., exon-skipping)	(54)
GSDMA	goblet cells	Caspase-3 cleavage	mitochondrial dysfunction and pyroptosis	mitochondrial dysfunction and pyroptosis	(55)

#### 2.3.1 GSDMD: central pyroptotic executor and therapeutic target

GSDMD represents the principal effector enabling pyroptotic membrane pore formation. Clinical analyses demonstrate that elevated levels of its N-terminal fragment (GSDMD-NT) in sputum directly correlate with neutrophilic airway inflammation and declining lung function (39). Pharmacologically, disulfiram, an FDA-approved drug, irreversibly modifies Cys191 on GSDMD, inhibiting pore formation. In murine models, disulfiram treatment diminishes IL-1β secretion and mitigates neutrophilic inflammation, highlighting a promising repurposing opportunity for neutrophilic asthma (56, 57).

#### 2.3.2 GSDME: mediator of epithelial pyroptosis and fibrosis in eosinophilic asthma

Gasdermin E (GSDME) is constitutively expressed in bronchial epithelial cells and can switch the mode of cell death from apoptosis to pyroptosis upon activation (58). In the context of severe eosinophilic asthma, GSDME plays a critical role in driving airway remodeling. Mechanistically, eosinophil-derived granzyme A (GzmA) can enter airway epithelial cells and activate caspase-3, which then cleaves GSDME. The resulting GSDME-NT fragments form pores, inducing pyroptotic death of epithelial cells (14). This process facilitates the release of profibrotic cytokines, such as transforming growth factor-beta 1 (TGF-β1), which activate fibroblasts and drive extracellular matrix deposition. Murine models deficient in GSDME reveal significantly reduced airway fibrosis and smooth muscle hypertrophy following prolonged allergen exposure, underlining GSDME’s specific role in linking eosinophilic inflammation to structural changes (59).

#### 2.3.3 GSDMB: genetic susceptibility and functional dualism

Gasdermin B (GSDMB), encoded in the asthma susceptibility locus 17q21, harbors SNPs that increase disease risk (60). It exhibits dual roles: the N-terminal domain enhances inflammation by potentiating caspase-1/4 activation of GSDMD, while the C-terminal domain binds sulfatides and suppresses NF-κB. Asthma-associated SNPs often generate truncated isoforms lacking the inhibitory domain, tilting the balance toward proinflammation (54). Its expression in airway epithelial and T-cell points to cell-specific roles in disease modulation (48).

#### 2.3.4 GSDMA: emerging role in viral exacerbations

Gasdermin A (GSDMA) has recently emerged as a mediator in severe asthma exacerbations, particularly linked to respiratory syncytial virus (RSV) infection. RSV fusion (F) protein can trigger mitochondrial dysfunction and oxidative stress, which specifically oxidizes GSDMA, inducing conformational changes that expose caspase-three cleavage sites (61). Caspase-3 cleavage liberates the pore-forming GSDMA-NT, leading to lytic cell death. Pathologically, GSDMA activation orchestrates the release of IL-33, amplifying ILC2 activation and type 2 inflammation, and facilitates mucus overproduction (62). This lytic process contributes critically to the pathophysiology of virus-induced asthma attacks (63).

## 3 Immune cells in asthma: beyond the Th2 paradigm

**Figure 3:**
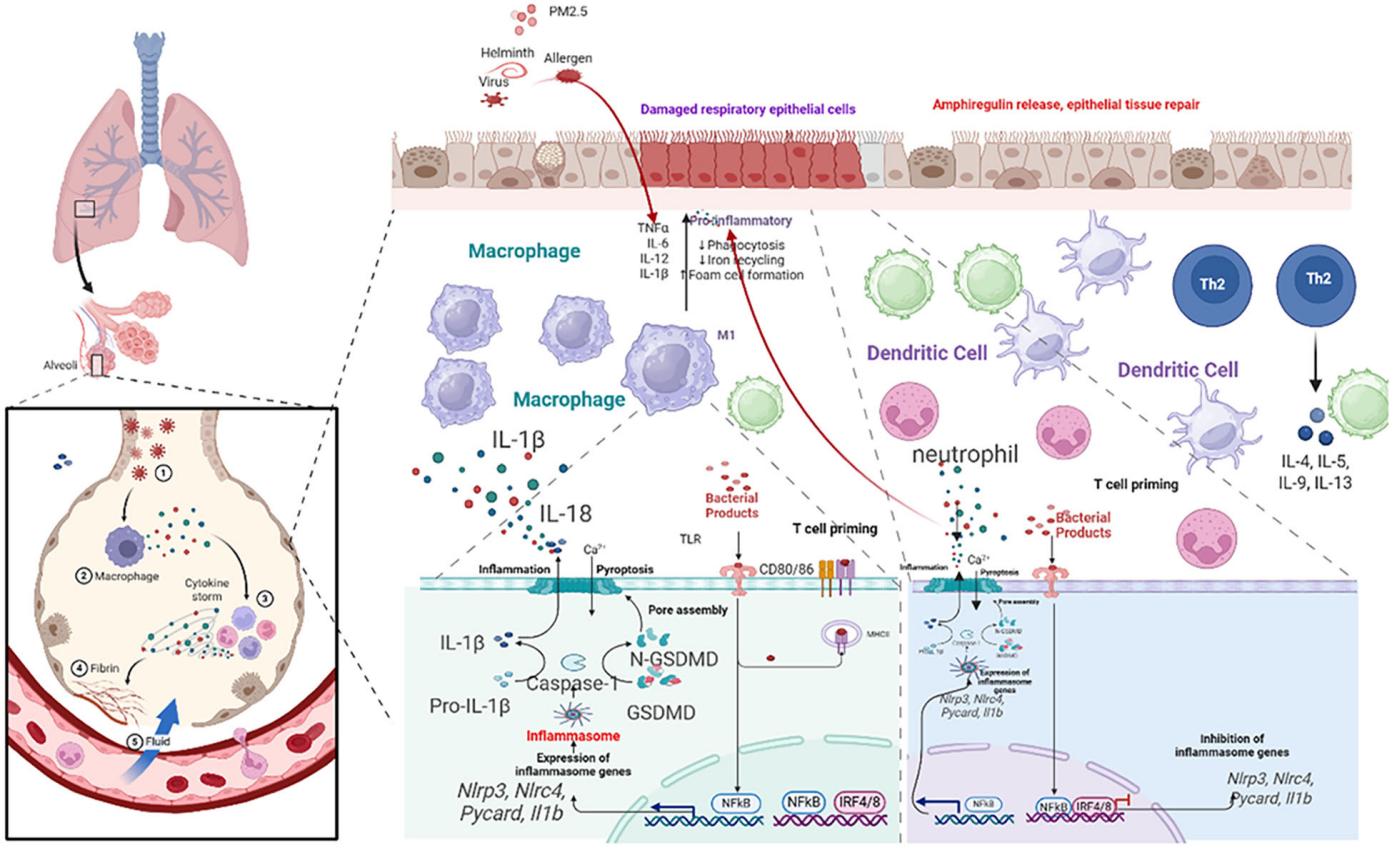
Schematic of airway epithelial pyroptosis in asthma. Illustrate triggers (PM, virus, allergen proteases) activating NLRP3 inflammasome via PRRs/damage, leading to caspase-1 activation, GSDMD cleavage/pore formation, release of IL-1β, IL-18, DAMPs, and consequences (neutrophil recruitment, ILC2 activation, barrier disruption, DAMP amplification).

The classical T-helper 2 (Th2) cell paradigm, while foundational to our understanding of allergic, eosinophilic asthma, is insufficient to explain the profound heterogeneity of the disease. The recognition of distinct endotypes—including Th2-high, Th2-low (neutrophilic or mixed), and paucigranulocytic asthma—demands a broader immunological perspective that incorporates the critical roles of innate immune cells and structural cells. Pyroptosis has emerged as a key inflammatory nexus, mediating chronic inflammation, AHR, and tissue remodeling through complex interactions that extend beyond classical Th2 pathways. This section delineates the pyroptosis-centric cross talk among various immune and structural cells, redefining their roles in asthma pathogenesis (summarized in **Table 4**, **Figure 3**).

**Table 4:** Non-Th2 immune cells in asthma: pyroptotic mechanisms and clinical implications.

Cell type	Classical asthma function	Pyroptosis activation pathway	Functional consequences	Clinical asthma phenotype association	References
Epithelial cells	Barrier integrity maintenance Alarmin release (TSLP/IL-25/IL-33)	Triggers: PM_2.5_/Viruses Mechanism: NLRP3 → Caspase-1 → GSDMD cleavage → IL-1β/IL-18 + DAMPs	ILC2/Th2 amplification Neutrophil recruitment Epithelial barrier rupture	Exacerbations Mixed/Neutrophilic asthma Severe disease	(64)
Eosinophils	Cytotoxic granule release (MBP/ECP/EPX/EDN) Th2 effector cells	Pathway 1: EETs → Macrophage AIM2 → Pyroptosis (IL-1β) Pathway 2: EPX → Direct GSDMD cleavage	IL-1β→Neutrophilia • IL-18→ILC2 hyperactivation • Direct epithelial damage	Severe eosinophilic asthma Epithelial remodeling Progressive disease	(65)
Mast cells	IgE/FcϵRI-mediated degranulation Release of histamine/tryptase/PGD_2_/CysLTs → AHR	Mechanism: Tryptase → PAR2 → NLRP3 potentiation → Epithelial pyroptosis	Barrier dysfunction IL-1β/IL-18 amplification; DAMP-mediated inflammation	AHR exacerbations Potential remodeling link	(66)
ILC2s	Alarmin-responsive IL-5/IL-13 production; Eosinophil/mucus regulation	Activation: IL-33 + ATP → IL-1β secretion Response: IL-18 receptor signaling	Autocrine ILC2 amplification; Synergistic type 2 inflammation; Innate-adaptive cross talk	Early-phase asthma Non-allergic triggers Severe type 2	(47)
M1 macrophages	Pro-inflammatory cytokine release (IL-1β/TNF-α); ROS/NO production	Triggers: LPS/Pollutants Pathway: Caspase-4/11 → GSDMD → NLRP3 → IL-1β/IL-18	Neutrophil chemotaxis; Steroid-resistant inflammation; Tissue destruction	Th2-low neutrophilic asthma Severe exacerbations Treatment resistance	(67)
M2 macrophages	Tissue repair (TGF-β/PDGF); Remodeling (MMPs); Th2 chemokine production	Note: Indirect modulation via Th2 cytokines Minimal direct pyroptosis involvement	Subepithelial fibrosis; Airway smooth muscle hyperplasia Angiogenesis	Chronic Th2-high asthma Irreversible obstruction Remodeling	(44)

### 3.1 Airway epithelial cells: the sentinels and primary target

The airway epithelium is the first line of defense, functioning as a dynamic immunological sentinel (68). Upon exposure to environmental insults (allergens, viruses, PM_2.5_), epithelial cells release alarmins (TSLP, IL-25, IL-33) that initiate type 2 immunity (64, 69–71). Crucially, these insults also directly induce epithelial pyroptosis. Activation of the NLRP3 or AIM2 inflammasome leads to caspase-1-mediated cleavage of GSDMD, forming plasma membrane pores that result in lytic cell death. This process releases preformed alarmins and mature forms of IL-1β and IL-18, alongside DAMPs (72, 73).

The consequences are twofold and endotype-defining: IL-1β drives neutrophilic inflammation and Th17 responses, characteristic of Th2-low asthma, while IL-18 potently synergizes with IL-33 to hyperactivate group 2 innate lymphoid cells (ILC2s), exacerbating eosinophilic inflammation in Th2-high asthma (74, 75). The uncontrolled release of DAMPs further establishes a feed-forward loop of inflammation and barrier dysfunction, positioning epithelial pyroptosis as a central initiating and amplifying mechanism in asthma (76).

### 3.2 Eosinophils: active orchestrators of pyroptotic inflammation

In severe eosinophilic asthma, eosinophils transcend their classical role as cytotoxic effector cells to become active drivers of pyroptosis through two distinct mechanisms:

EETosis-driven macrophage pyroptosis: Activated eosinophils release eosinophil extracellular traps (EETs) (77). The DNA component of these EETs acts as a DAMP, sensed by the AIM2 inflammasome in airway macrophages (78). This triggers caspase-1 activation, macrophage pyroptosis, and the release of IL-1β and IL-18, thereby linking local eosinophilia to broader, systemic innate immune activation.

Direct epithelial pyroptosis: Eosinophil peroxidase (EPX) can directly cleave and activate GSDMD in airway epithelial cells, bypassing the canonical caspase machinery. This induces pyroptotic barrier disruption and alarmin release, creating a direct pathogenic link between eosinophil infiltration and epithelial damage (79).

This dual capacity of eosinophils to instigate pyroptosis in both immune and structural compartments underscores their role as critical orchestrators in severe, remodeling-associated disease and reveals novel therapeutic targets within the pyroptotic network.

### 3.3 Mast cells and ILC2s: innate amplifiers of pyroptotic cascades

Mast cells are strategically localized beneath the epithelium. Upon IgE-mediated activation, they release tryptase, which engages protease-activated receptor-2 (PAR2) on epithelial cells, potentiating NLRP3 inflammasome assembly and lowering the threshold for epithelial pyroptosis (65). Type 2 innate lymphoid cells (ILC2s) are pivotal rapid responders. While not major pyroptosis executioners, their activity is profoundly modulated by pyroptotic outputs. ILC2s can produce IL-1β, creating an autocrine loop, and IL-18 released from pyroptotic cells synergizes powerfully with IL-33 to boost ILC2 activation, explaining how non-allergic triggers can provoke robust type 2 inflammation (80).

### 3.4 Macrophages: polarized regulators and effectors of pyroptosis

**Figure 4:**
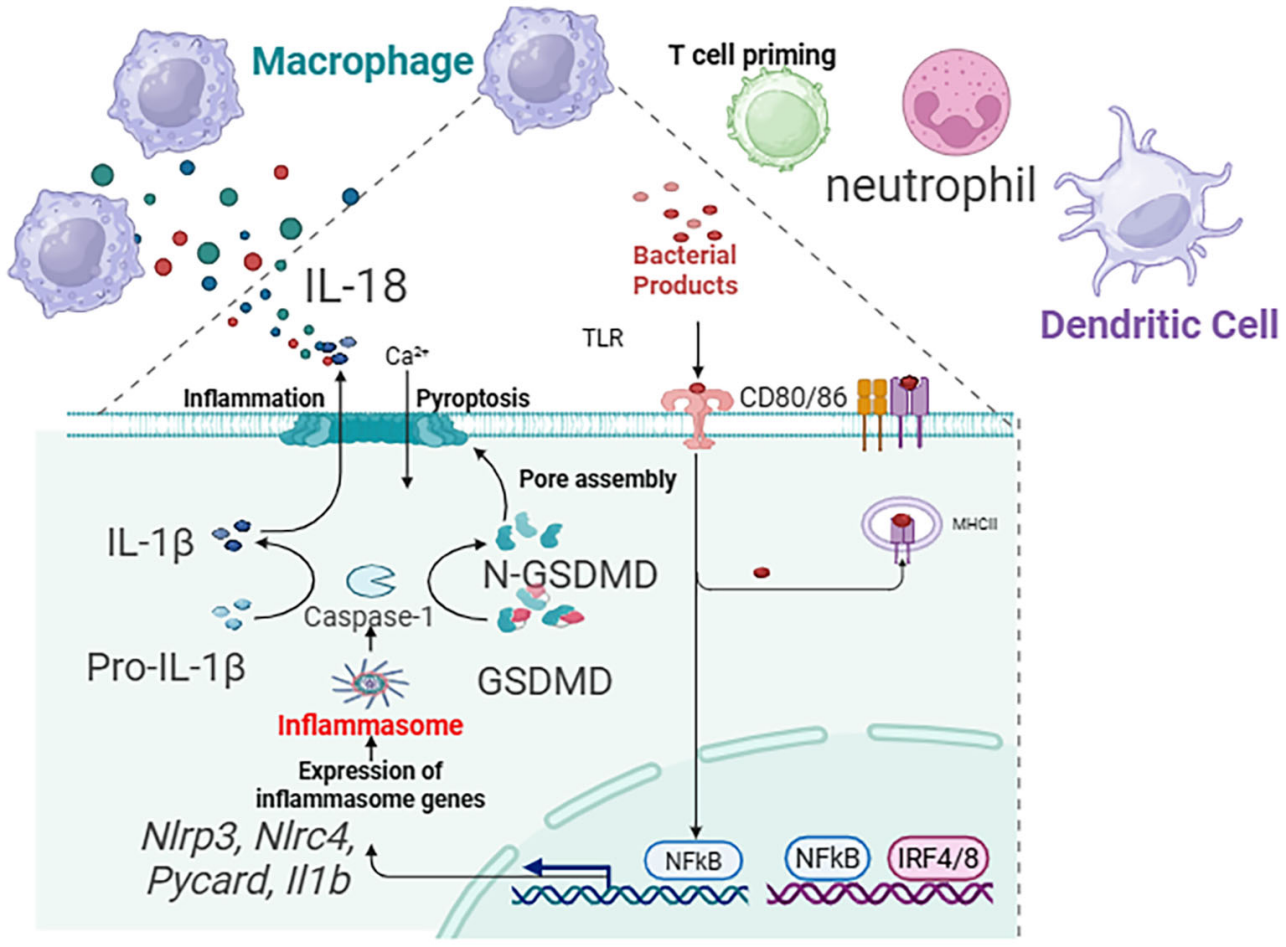
Summary diagram that integrates the roles of epithelial cells, eosinophils, macrophages, and ILC2s within the pyroptosis network across asthma endotypes.

As the most abundant immune cells in the healthy lung, macrophages exhibit remarkable plasticity, and their role in pyroptosis is phenotype-dependent. M2 Macrophages dominate in Th2-high asthma, driven by IL-4 and IL-13. They are not primary agents of pyroptosis but are crucial for its long-term consequences (81). Through the release of TGF-β, PDGF, and matrix-modulating enzymes (MMPs, TIMPs), M2 macrophages drive the pathological remodeling—subepithelial fibrosis, smooth muscle hyperplasia—that characterizes chronic, treatment-resistant Th2-high asthma. Their activity is sustained by the Th2 cytokines amplified by pyroptosis-initiated circuits (82). M1 Macrophages are key players in Th2-low, neutrophilic asthma. They are principal effectors of the non-canonical pyroptosis pathway. Environmental triggers like bacterial LPS (from colonizing pathogens) or pollutants activate caspase-4/5 in human macrophages, leading to GSDMD cleavage and pyroptosis. The ensuing pore formation and K^+^ efflux often activate the canonical NLRP3 inflammasome, creating an amplification loop. The resultant massive release of IL-1β is a master driver of neutrophilic inflammation, upregulating chemokines (CXCL1, CXCL8) and promoting Th17 differentiation. Concurrently, released IL-18 and DAMPs intensify the inflammatory milieu. This pathway is a core mechanism underlying the steroid resistance and frequent exacerbations in severe neutrophilic asthma, making macrophages a prime therapeutic target for this difficult-to-treat endotype. A summary diagram integrating the roles of key cells within the pyroptosis network across asthma endotypes is presented in **Figure 4**.

## 4 The role of pyroptosis in asthma: from biomarkers to therapeutic implications

Pyroptosis is not merely a bystander but an active driver that exacerbates asthma severity, increases exacerbation risk, and underlies therapeutic resistance. This section delves into the translational aspects of pyroptosis, highlighting validated and emerging biomarkers, insights from cutting-edge transcriptomic technologies, key genetic regulators, and the consequent therapeutic strategies that are shaping a new era in asthma management. A summary of therapeutic strategies targeting pyroptosis is provided in **Table 5**.

**Table 5:** Therapeutic targeting of pyroptosis in asthma.

Therapeutic target	Candidate agent(s)	Mechanism of action	Current stage of development	Potential asthma context/endotype	References
NLRP3 Inflammasome	MCC950, IZD334 (mAb)	Inhibits NLRP3 oligomerization	Preclinical, Phase I	Severe Asthma (Th2-high & Th2-low)	(94)
Caspase-1	VX-765 (Belnacasan)	Irreversible caspase-1 inhibitor	Phase II (discontinued; inhaled precl.)	Severe, inflammasome-driven asthma	(95)
GSDMD Pore	Disulfiram, Necrosulfonamide	Covalently modifies GSDMD to block pore formation	Phase 2a (NCT04808966), Preclinical	Neutrophilic, Steroid-resistant asthma	(96)
IL-1β	Canakinumab (mAb)	Neutralizes IL-1β	Phase II (NCT04025554)	Severe Neutrophilic Asthma	(97)
Non-canonical caspases	Emricasan (pan-caspase)	Inhibits caspases-1, -3, -4, -5	Preclinical	Viral exacerbations, Neutrophilic inflammation	(98)
Gene silencing (GSDMD/E)	siRNA (LNP/formulated)	Degrades target mRNA	Preclinical	Endotype-specific (e.g., GSDME for remodeling)	(99)
Gene editing (NLRP3)	CRISPR Base Editing	Corrects gain-of-function mutations (e.g., Q705K)	Preclinical (proof-of-concept)	Patients with specific hyperactive NLRP3 variants	(100)
Bispecific antibody	AZD5423 (preclinical)	Simultaneously neutralizes GSDMD and IL-1β	Preclinical	Mixed neutrophilic-eosinophilic inflammation	(101)

### 4.1 Validated pyroptosis biomarkers in asthma: informing endotype-driven therapy

The clinical translation of pyroptosis biology hinges on the identification of robust, measurable biomarkers. These indicators, detectable in serum, sputum, and bronchoalveolar lavage fluid (BALF), provide a dynamic window into disease activity and therapeutic response, paving the way for precision medicine.

#### 4.1.1 Interleukin-18

As a key product of inflammasome activation, IL-18 is consistently elevated in asthma, with particularly high levels observed in neutrophilic and severe phenotypes. Elevated serum IL-18 correlates with increased exacerbation risk and steroid resistance. It serves a dual role as both a marker of active pyroptotic inflammation and a promising therapeutic target itself (83).

#### 4.1.2 Gasdermin D N-terminal fragment

The cleaved, active form of gasdermin D N-terminal fragment (GSDMD) is a direct executor of pyroptosis. Quantifiable GSDMD-N in induced sputum offers a precise measure of ongoing pyroptotic activity in the airways. Its levels correlate with neutrophilic inflammation and declining lung function. Crucially, interventions like disulfiram, which targets GSDMD, lead to a measurable reduction in sputum GSDMD-N, validating its utility as a pharmacodynamic biomarker (84).

#### 4.1.3 Caspase-1 activity

As the core effector of the canonical inflammasome pathway, caspase-1 activity is a proximal marker of inflammasome activation. Increased caspase-1 activity in immune cells or bronchial tissue is a hallmark of steroid-resistant asthma, often triggered by viral infections or pollutants, implicating it in the pathogenesis of severe exacerbations (16).

#### 4.1.4 Gasdermin E N-terminal fragment

Emerging as a biomarker for structural disease, gasdermin E N-terminal fragment (GSDME-N) is implicated in airway remodeling. Its elevation, particularly in the context of severe eosinophilic asthma, is associated with epithelial damage driven by the eosinophil granzyme A-caspase-3 axis and correlates with progressive fibrosis and fixed airflow obstruction (85).

Clinical integration: The combinatorial use of these biomarkers can stratify patients into pathway-specific endotypes. For instance, a profile of high sputum GSDMD-N and serum IL-18 defines a pyroptosis-driven neutrophilic endotype amenable to NLRP3 or IL-1β blockade. Conversely, elevated GSDME-N in a Th2-high context may identify patients with remodeling-prone disease who could benefit from anti-IL-5/IL-13 biologics combined with GSDME-targeted strategies.

### 4.2 Insights from single-cell and spatial transcriptomics: mapping the pyroptotic niche

Advanced omics technologies have revolutionized our understanding of asthma heterogeneity by defining the cellular and spatial context of pyroptosis (86).

#### 4.2.1 Cellular landscapes of pyroptosis

Single-cell RNA sequencing (scRNA-seq) of bronchial epithelial cells from severe asthma (SA) patients reveals a distinct pyroptotic signature. Key genes such as GSDMD, CASP4, and NLRP3 are significantly upregulated in ciliated cells, while GSDME is elevated in goblet cells within remodeling niches. This cell type–specific expression pattern links pyroptosis directly to both inflammation and structural alterations (87).

#### 4.2.2 The pyroptosis signature score

To quantify this, researchers have developed a PSS based on the aggregate expression of pyroptosis-related genes. The PSS robustly distinguishes SA from mild asthma (MA) and serves as a powerful predictive biomarker for disease severity, potentially guiding the selection of patients for pyroptosis-targeted trials (88).

#### 4.2.3 Spatial architecture of inflammation

Spatial transcriptomics has localized pyroptotic epithelial cells to specific fibrotic niches within the airway wall. These niches are co-enriched with activated fibroblasts and IL-17–producing T cells, providing direct visual evidence that localized pyroptosis fosters a pro-fibrotic and type 17-inflammatory microenvironment, thereby driving remodeling (89).

#### 4.2.4 Immune cell contributions revealed

scRNA-seq has identified non-classical monocytes (CD14^+^CD16^+^) in severe asthma lungs that exhibit primed inflammasome states (high AIM2, CASP1). Furthermore, it has confirmed the activation of the GZMA–caspase-3–GSDME axis within eosinophils themselves, illustrating a complex cellular choreography where multiple cell types contribute to the pyroptotic network (90).

### 4.3 Genetic and epigenetic modulators of pyroptosis in asthma

Individual susceptibility to pyroptosis-driven inflammation is shaped by genetic and epigenetic factors.

#### 4.3.1 The 17q21 locus and GSDMB

This major asthma risk locus contains the GSDMB gene. The risk allele rs7216389 is an expression quantitative trait locus (eQTL) that increases GSDMB expression, potentiating caspase-4–dependent epithelial pyroptosis and barrier dysfunction. This provides a mechanistic link between the most significant genetic risk factor for asthma and the pyroptotic pathway.

#### 4.3.2 NLRP3 Q705K polymorphism

This gain-of-function variant in NLRP3 leads to constitutive inflammasome activation and is associated with severe, adult-onset asthma. Identifying carriers of this and similar variants could enable preemptive targeting of the NLRP3 pathway.

#### 4.3.3 Epigenetic regulation

Epigenetic mechanisms fine-tune pyroptotic responses. Hypomethylation of the ASC (PYCARD) promoter enhances inflammasome assembly. Conversely, microRNAs like miR-223-3p, which suppresses NLRP3 translation, are downregulated in severe asthma, permitting inflammasome hyperactivation. These modulators represent novel targets for intervention.

### 4.4 Therapeutic implications: from preclinical models to human biology

Proof-of-concept studies in murine models provide strong rationale for targeting pyroptosis (91).

#### 4.4.1 NLRP3 inhibition

The selective inhibitor MCC950 significantly reduces IL-1β levels, eosinophilic infiltration, and AHR in allergen-challenge models, validating NLRP3 as a druggable target for Th2-high inflammation (92).

#### 4.4.2 Gasdermin D targeting

Disulfiram, an FDA-approved drug for alcohol dependence, has been repurposed to inhibit GSDMD pore formation by irreversibly modifying its Cys191 residue. In chronic house dust mite (HDM) models, disulfiram administration resulted in decreased airway remodeling indicators, including a 54% reduction in collagen I deposition, and improved AHR (57). This highlights the potential of targeting GSDM pathways in asthma management, particularly in patients exhibiting pyroptosis-related inflammation.

#### 4.4.3 Silencing gasdermin expression

siRNA-mediated knockdown of GSDME reduces goblet cell metaplasia and TGF-β1-driven fibrosis, highlighting its specific role in remodeling and its potential as a target in eosinophilic asthma (93).

## 5 Therapeutic targeting of pyroptosis in asthma: a precision medicine approach

The persistent burden of severe asthma, particularly in patients’ refractory to current standard of care, demands novel strategies that target upstream pathogenic drivers. Pyroptosis, as a central amplifier of inflammation, barrier dysfunction, and remodeling, represents a paradigm-shifting therapeutic frontier. This section comprehensively examines the evolving landscape of pyroptosis-targeted therapeutics, from pharmacological inhibitors to advanced modalities, emphasizing a precision medicine framework.

### 5.1 Pharmacological inhibition of core machinery

#### 5.1.1 NLRP3 inflammasome inhibitors

MCC950, a potent and selective small-molecule inhibitor, has demonstrated remarkable efficacy in preclinical models, reducing AHR and inflammation. Its synergy with corticosteroids by mitigating IL-1β–driven JAK-STAT activation is a key advantage for overcoming steroid resistance (102).

#### 5.1.2 Caspase-1 inhibitors

VX-765 showed efficacy in animal models but its systemic use was limited by hepatotoxicity. This has spurred the development of inhaled formulations and nanoparticle-encapsulated versions to achieve local lung efficacy while minimizing systemic exposure (103).

#### 5.1.3 Gasdermin-D pore blockers

Direct inhibition of the terminal effector holds immense promise. Disulfiram has rapidly moved into clinical trials for asthma based on compelling preclinical and early clinical data showing reduced exacerbations and sputum GSDMD-N. Next-generation covalent inhibitors like oridonin offer even greater potency (104).

### 5.2 Biologics and cytokine-targeted strategies

#### 5.2.1 Cytokine neutralization

Monoclonal antibodies against IL-1β (Canakinumab) and IL-18 (GSK1070806) are in Phase II trials for severe neutrophilic and Th2-low asthma, respectively, providing a direct strategy to quench the inflammatory output of pyroptosis.

#### 5.2.2 Engineered biologics

Novel approaches include anti-GSDMD nanobodies for inhalation and monoclonal antibodies (e.g., IZD334) that target the NLRP3 inflammasome itself, offering upstream inhibition of the canonical pathway.

### 5.3 Gene silencing and editing for precision targeting

RNA-based therapies and gene editing offer highly specific, potentially long-lasting solutions.

#### 5.3.1 siRNA technology

Lipid nanoparticles (LNPs) encapsulating siRNA against GSDMD or GSDME have shown efficacy in preclinical models, achieving significant knockdown in the lung and attenuating exacerbations and remodeling. Targeted delivery systems (e.g., peptide-guided nanoparticles) enhance epithelial specificity.

#### 5.3.2 CRISPR gene editing

For patients with defined genetic hyperactivation, such as the NLRP3 Q705K variant, CRISPR-mediated base editing can permanently correct the mutation at the DNA level in patient-derived cells, representing the ultimate form of precision medicine.

### 5.4 Rationale and evidence for combination therapies

Given the complexity of asthma, combination strategies are essential. Pyroptosis inhibitors can complement existing therapies:

#### 5.4.1 With biologics

A GSDMD inhibitor can augment the efficacy of an anti-IL-5 biologic (e.g., mepolizumab) by addressing the epithelial damage and remodeling that eosinophil depletion alone does not.

#### 5.4.2 With corticosteroids

By targeting IL-1β–driven steroid resistance, a pyroptosis inhibitor can restore sensitivity to inhaled corticosteroids, allowing for lower steroid doses.

#### 5.4.3 Dual pyroptosis pathway inhibition

Combining an NLRP3 inhibitor (canonical) with an agent that reduces caspase-4 expression (non-canonical) provides broad coverage against multiple triggers.

Clinical trials evaluating such combinations (e.g., MCC950 with benralizumab) are already underway, signaling a shift towards mechanism-based, combination immunotherapy.

### 5.5 Advanced delivery systems and repurposing

Targeted delivery: To maximize safety and efficacy, advanced delivery systems are being developed. These include inhaled nanoparticles for macrophage-specific NLRP3 inhibition and prodrugs activated only by disease-specific enzymes (e.g., MMP-12) in the remodeled airway, minimizing systemic effects.

#### 5.5.1 Drug repurposing

Beyond disulfiram, other agents like tranilast (an anti-allergic drug with NLRP3 inhibitory properties) and natural compounds like andrographolide and oridonin offer a faster track to clinical application.

### 5.6 Future research trajectories and frontiers

Several exciting frontiers are emerging:

**Microbiome-metabolite axis**: Metabolites like butyrate from the gut microbiome can epigenetically suppress NLRP3 via miR-223, opening avenues for dietary or probiotic interventions.

**Bispecific antibodies:** Molecules like the preclinical candidate AZD5423, which simultaneously neutralizes GSDMD and IL-1β, show superior efficacy in models of mixed inflammation by targeting both the executor and a key cytokine.

**Overcoming PANoptosis:** Recognizing the cross talk between cell death pathways (PANoptosis), future therapies may need to co-target key regulators like ZBP1 to prevent compensatory inflammation.

**Digital twins and personalized models:** The future may lie in creating patient-specific computational models that simulate individual pyroptosis pathways to predict optimal therapeutic combinations, truly ushering in the era of personalized asthma management.

### 5.7 Challenges and future research trajectories

Despite the immense promise, several hurdles must be overcome for the successful clinical implementation of pyroptosis-targeted therapies in asthma. Systemic, non-selective inhibition of core pyroptotic components like GSDMD carries the risk of compromising innate immune defenses against bacterial, viral, and fungal pathogens. Developing delivery systems that target specific lung cell types (epithelium, specific immune subsets) involved in asthma pathogenesis while sparing systemic immunity is a critical ongoing challenge. The successful implementation of pyroptosis-targeted therapies, especially expensive biologics and advanced modalities, hinges on identifying reliable, accessible biomarkers for patient stratification and treatment monitoring. The current lack of validated, point-of-care assays for key markers like active GSDMD fragments (GSDMD-N), specific IL-18 isoforms, or inflammasome activation signatures impedes precision medicine approaches. Robust validation of proposed biomarkers (e.g., serum GSDME-N, sputum GSDMD-N) in large, diverse patient cohorts is urgently needed.

Cell death pathways exhibit significant cross talk. Inhibition of pyroptosis alone might be insufficient if compensatory mechanisms like necroptosis (another lytic pathway) are activated—a concept termed PANoptosis. This may necessitate co-targeting strategies (e.g., combining necrosulfonamide with necroptosis inhibitors like necrostatin-1), adding complexity to therapeutic regimens. Understanding the dominant cell death pathways in specific asthma endotypes and exacerbation triggers is vital.

The following several exciting emerging fields are worth exploring for the treatment of asthma by regulating pyroptosis. The airway and gut microbiome influence inflammation. The airway and gut microbiome influence inflammation. Metabolites like butyrate, produced by commensals such as Faecalibacterium prausnitzii, can upregulate microRNA-223 (miR-223), which directly suppresses NLRP3 mRNA translation, offering a potential dietary or probiotic strategy to dampen pollutant-induced pyroptosis (105). Furthermore, emerging techniques such as spatial transcriptomics are poised to precisely map pyroptotic cells within the complex architecture of the asthmatic airway, identifying critical cellular niches that drive disease (106). The application of novel screening platforms could accelerate the discovery of next-generation, highly specific GSDM inhibitors. Additionally, a deeper understanding of PANoptosis, an integrated inflammatory cell death pathway, is crucial, as targeting key regulators like ZBP1 may be necessary to overcome compensatory cell death mechanisms and achieve maximal therapeutic efficacy in severe asthma (106). Developing patient-specific computational models that simulate individual pyroptosis pathway activation in response to triggers and therapies could revolutionize treatment selection. These “digital twins” could predict optimal combinations and sequencing of pyroptosis inhibitors, biologics, and corticosteroids for maximal efficacy and minimal toxicity.

### 5.8 Frontiers in targeted therapeutics: gene editing, bispecific antibodies, and microbiome modulation

The rapid development of molecular and cellular therapeutics has ushered in a new era of precision medicine aimed at modulating key inflammatory pathways underlying chronic diseases such as asthma, autoinflammatory syndromes, and fibrosis. Recent preclinical and translational studies highlight promising advances in gene editing technologies, novel bispecific antibodies, and microbiome-driven epigenetic regulation that target pivotal components, including the NLRP3 inflammasome, GSDMD, and IL-1β. Genetic mutations in NLRP3, a critical inflammasome sensor, have been implicated in various autoinflammatory conditions characterized by excessive caspase-1 activation, IL-1β release, and pyroptotic cell death. Among these, the Q705K missense mutation induces a gain-of-function phenotype leading to aberrant inflammasome activation (107). A recent preclinical study demonstrates the successful correction of the NLRP3 Q705K mutation using CRISPR-mediated adenine base editing (ABE) in patient-derived macrophages (108). This approach harnesses the precision of CRISPR base editors to catalyze a targeted A•T to G•C nucleotide transition without inducing double-strand breaks, thus minimizing genomic instability. Functional assays confirmed restoration of wild-type inflammasome regulation, with markedly reduced caspase-1 cleavage and IL-1β secretion upon canonical stimulation. *In-vivo* administration of the ABE system in murine models carrying the Q705K knock-in mutation resulted in attenuation of systemic inflammation and tissue pathology. These findings underscore the transformative potential of CRISPR base editing as a durable therapeutic strategy to correct pathogenic inflammasome mutations at the DNA level, offering a new paradigm beyond transient cytokine blockade. Further optimization of delivery vectors and off-target profiling will be critical to advance clinical translation.

Monoclonal antibodies have revolutionized treatment for inflammatory diseases; however, clinical phenotypes often involve complex immune cell interplay beyond single cytokine targets. To address this, bispecific antibodies capable of simultaneously modulating multiple inflammatory mediators are emerging as promising next-generation therapeutics. A recently reported candidate, AZD5423, is a bispecific antibody that concurrently neutralizes GSDMD—the terminal effector of pyroptosis— and IL-1β, a pivotal cytokine driving inflammasome-mediated inflammation (109). Preclinical evaluation in murine models of mixed neutrophilic and eosinophilic airway inflammation demonstrated superior efficacy of AZD5423 over monospecific antibodies. Mechanistically, blockade of GSDMD pores curtailed pyroptotic cell lysis and release of damage-associated molecular patterns (DAMPs), thereby suppressing the recruitment and activation of neutrophils. Simultaneously, IL-1β neutralization dampened type 2 immune responses characterized by eosinophil infiltration and Th2 cytokine production. The dual-targeting strategy showed a marked reduction in AHR, mucus overproduction, and tissue remodeling markers. Importantly, AZD5423 was well-tolerated without compromising host defense in infectious challenge models. These data highlight bispecific antibodies as a compelling approach to address the multifaceted immune phenotypes in chronic inflammatory diseases by interrupting interconnected pathogenic pathways.

The gut-lung axis embodies a critical conduit through which microbiota-derived metabolites influence pulmonary immunity and inflammation (110). Notably, short-chain fatty acids (SCFAs), particularly butyrate, exert profound immunomodulatory effects via epigenetic mechanisms. A recent study published in Microbiome elucidated that butyrate supplementation attenuates NLRP3 inflammasome activation in airway epithelial cells and macrophages by inhibiting histone deacetylase 3 (HDAC3) (111). HDAC3 inhibition resulted in chromatin remodeling that favored the expression of microRNA-223 (miR-223), a critical posttranscriptional regulator that targets NLRP3 mRNA for degradation (112). Functional validation revealed that enhanced miR-223 levels consequent to butyrate treatment lowered NLRP3 protein abundance, thus repressing caspase-1 activation and downstream IL-1β maturation. In murine models of allergen- and virus-induced airway inflammation, butyrate administration reduced neutrophilic inflammation and airway hyperreactivity, effects that were abrogated upon miR-223 knockdown (113). This study underscores a novel microbiome-metabolite-epigenetic axis modulating innate immune sensors, providing a compelling rationale for dietary or microbiota-targeted interventions to control inflammasome-driven diseases.

The integration of gene editing, innovative biologics, and microbiome-based therapies represents a multidisciplinary frontier in targeted treatment of inflammasome-related inflammatory diseases. CRISPR base editing offers the promise of precise, permanent correction of pathogenic genetic variants such as NLRP3 Q705K, potentially transforming management of hereditary autoinflammatory syndromes. Bispecific antibodies like AZD5423 exemplify rational design of multifunctional therapeutics that concurrently mitigate pyroptotic cell death via GSDMD blockade and cytokine-driven inflammation through IL-1β neutralization, addressing complex immune phenotypes such as mixed neutrophilic-eosinophilic asthma.

Simultaneously, microbiome-derived metabolites, including butyrate, provide an accessible avenue to epigenetically reprogram innate immune responses and ameliorate inflammasome hyperactivation by inducing miR-223 expression and suppressing NLRP3 transcription. These findings advocate for the therapeutic exploitation of the gut-lung axis in chronic inflammatory conditions. Future directions will necessitate combined strategies integrating genetic, immunologic, and metabolic interventions, along with personalized biomarker-guided patient stratification to maximize efficacy and minimize adverse effects. The continuing evolution of delivery technologies, safety validation, and mechanistic insights will propel these targeted therapies closer to clinical reality, promising more effective and durable treatment options for patients burdened by inflammasome-associated airway diseases and systemic autoinflammation.

Therapeutic targeting of pyroptosis represents a transformative strategy for severe asthma, moving beyond symptom control towards potential disease modification. The arsenal is diverse and rapidly evolving. While challenges regarding targeted delivery, biomarker validation, and pathway redundancy remain, the strategic integration of pyroptosis endotyping with innovative therapeutics holds the key to unlocking personalized and highly effective treatments for the most vulnerable asthma patients.

## 6 Challenges and future directions in pyroptosis-targeted asthma therapeutics

The burgeoning recognition of pyroptosis as a pivotal mechanism in asthma pathogenesis has opened a promising new frontier for therapeutic intervention. However, the journey from mechanistic understanding to clinical application is fraught with challenges that reflect the inherent complexity of both the cell death pathway and asthma itself. This section delineates the principal hurdles and charts a course for future research, emphasizing a precision medicine approach to unlock the full potential of pyroptosis-modulating therapies.

### 6.1 Key challenges in clinical translation

#### 6.1.1 Therapeutic specificity and host defense

A paramount concern is the risk of systemic immunosuppression. Global inhibition of core pyroptotic components like GSDMD or NLRP3 could compromise innate immunity against bacterial, viral, and fungal pathogens, given their critical role in host defense. The challenge lies in achieving cell-type and context-specific inhibition, sparing systemic immunity while quelling pathogenic airway inflammation.

#### 6.1.2 Biomarker validation and clinical deployment

While promising biomarkers like GSDMD-N and IL-18 have been identified, their transition to routine clinical practice is impeded. There is a pressing need for the development of rapid, point-of-care assays (e.g., lateral flow tests for GSDMD-N) and the robust validation of these biomarkers in large, diverse, and longitudinal patient cohorts to establish standardized cut-off values and link them definitively to treatment responses.

#### 6.1.3 Pathway redundancy and PANoptosis

The inflammatory cell death landscape is not monolithic. Inhibition of pyroptosis alone may be insufficient if compensatory mechanisms, such as necroptosis or apoptosis, are activated—a convergence point known as PANoptosis. In severe, refractory asthma, this interplay may necessitate complex co-targeting strategies (e.g., simultaneously inhibiting GSDMD and the necroptosis mediator MLKL), adding layers of complexity to therapeutic development.

#### 6.1.4 Targeted drug delivery

The efficacy and safety of pyroptosis inhibitors are inextricably linked to delivery. Achieving high local concentrations in specific lung cell types (e.g., epithelium for GSDME inhibition, macrophages for NLRP3 blockade) while minimizing systemic exposure is crucial. Advancements in nanocarriers and inhaled formulations are vital to overcome this pharmacokinetic challenge.

### 6.2 Strategic future directions

#### 6.2.1 To overcome these challenges, a multi-pronged research strategy is essential

Precision Targeting of the Airway Niche: Future efforts must focus on advanced delivery platforms. This includes the development of:

#### 6.2.2 Cell-specific nanocarriers

Using ligands that target receptors abundant on specific lung cells (e.g., ligands for alveolar macrophages or stressed epithelium).

#### 6.2.3 Conditionally active prodrugs

Designing therapeutics activated only in the inflammatory microenvironment of the asthmatic airway (e.g., by enzymes like MMP-12).

#### 6.2.4 Inhaled biologics and siRNAs

Optimizing formulations for pulmonary delivery of anti-GSDMD nanobodies or gene-silencing agents to achieve local efficacy.

#### 6.2.5 Integrating multi-omics for endotype refinement

Future studies must leverage single-cell and spatial transcriptomics not just for discovery, but for clinical stratification. Defining a “pyroptosis signature” from bronchial biopsies or even less invasively, from sputum cells, could identify the subset of patients most likely to respond to these targeted therapies. This will move the field beyond broad Th2/Th2-low classifications towards pathway-driven endotyping.

### 6.3 Confronting PANoptosis and the microbiome:

#### 6.3.1 PANoptosis therapeutics

Research should prioritize identifying and targeting master regulators of PANoptosis complexes, such as ZBP1. This upstream approach may be more effective than co-inhibiting multiple downstream death effectors.

#### 6.3.2 Modulating the microbiome-pyroptosis axis

The role of the airway and gut microbiome in shaping pyroptotic responses is a nascent but critical area. Investigating how microbial metabolites (e.g., short-chain fatty acids like butyrate) or specific pathobionts (e.g., Haemophilus influenzae) influence inflammasome priming could lead to novel interventions, such as probiotics or prebiotics, to dampen pyroptotic hyperresponsiveness.

#### 6.3.3 Understanding asthma inception and lifelong course

Emerging evidence suggests pyroptosis may be programmed early in life. Future research must utilize longitudinal birth cohorts to investigate how prenatal exposures (e.g., PM2.5 inducing epigenetic changes in GSDMD) and severe early-life viral infections (e.g., RSV activating caspase-4) prime the immune system for subsequent asthma development. This “asthma inception” paradigm could open avenues for very early preventative strategies.

#### 6.3.4 Developing predictive in-silico models

The integration of multi-omics data, clinical parameters, and pharmacokinetic-pharmacodynamic (PK/PD) models can lead to the creation of “digital twins” for individual patients. These computational models could simulate responses to various pyroptosis-targeted therapies, predicting optimal drug combinations and dosing regimens to maximize efficacy and minimize toxicity in a truly personalized manner.

## 7 Conclusions

In summary, this review consolidates the paradigm that pyroptosis is not a peripheral phenomenon but a central pathogenic axis in asthma, critically integrating environmental triggers with dysregulated immune responses. We have delineated a cohesive model wherein distinct pyroptotic pathways drive specific asthma endotypes: allergen-driven, NLRP3-GSDMD/GSDME-mediated circuits predominantly fuel the type 2 inflammation and remodeling of Th2-high/eosinophilic asthma, whereas pollutant- and virus-activated, non-canonical caspase-4/5-AIM2 axes propel the neutrophilic inflammation and steroid resistance characteristic of Th2-low asthma. The emergent concept of PANoptosis provides a plausible mechanistic framework for the profound treatment recalcitrance observed in the most severe forms of the disease.

The translational implications of this knowledge are profound. The repertoire of pyroptosis-related biomarkers—from IL-18 to active GSDM fragments—holds immense potential to refine patient stratification, moving clinical practice towards a pathway-based classification system. Correspondingly, the therapeutic pipeline is rapidly expanding, encompassing an arsenal of small-molecule inhibitors, biologics, and advanced gene-targeting modalities designed to precisely intercept this inflammatory cell death cascade at multiple nodes.

Looking forward, the successful clinical implementation of pyroptosis-targeted therapies hinges on confronting key challenges: ensuring localized delivery to safeguard systemic host defense, validating robust biomarkers for patient selection, and understanding the interplay within the broader cell death network. By embracing these challenges as opportunities and leveraging cutting-edge technologies from spatial transcriptomics to in silico modeling, the field is poised to usher in a new era of precision medicine for asthma. Targeting pyroptosis represents a fundamental shift from managing symptoms to potentially modifying the disease course, offering hope for transformative outcomes for the millions worldwide affected by severe and refractory asthma.
